# Residential Greenspace and Urban Adolescent Substance Use: Exploring Interactive Effects with Peer Network Health, Sex, and Executive Function

**DOI:** 10.3390/ijerph18041611

**Published:** 2021-02-08

**Authors:** Jeremy Mennis, Xiaojiang Li, Mahbubur Meenar, J. Douglas Coatsworth, Thomas P. McKeon, Michael J. Mason

**Affiliations:** 1 Department of Geography and Urban Studies, Temple University, Philadelphia, PA 19122, USA; xiaojiang.li@temple.edu (X.L.); mckeont@temple.edu (T.P.M.); 2 Department of Geography, Planning, and Sustainability, Rowan University, Glassboro, NJ 08028, USA; meenar@rowan.edu; 3 Center for Behavioral Health Research, University of Tennessee, Knoxville, TN 37996, USA; dcoatswo@utk.edu (J.D.C.); mmason29@utk.edu (M.J.M.)

**Keywords:** greenspace, mental health, substance use, peers, executive function, environmental susceptibility, differential susceptibility, adolescents

## Abstract

While urban greenspace is increasingly recognized as important to mental health, its role in substance use is understudied. This exploratory study investigates the interaction of greenspace with peer network health, sex, and executive function (EF) in models of substance use among a sample of disadvantaged, urban youth. Adolescents and their parents were recruited from a hospital in the mid-Atlantic region of the U.S. Residential greenspace at the streetscape level was derived from analysis of Google Street View imagery. Logistic regression models were used to test the moderating effect of greenspace on the association between peer network health and substance use, as well as additional moderating effects of sex and EF. The significant negative association of peer network health with substance use occurred only among youth residing in high greenspace environments, a moderating effect which was stronger among youth with high EF deficit. The moderating effect of greenspace did not differ between girls and boys. Greenspace may play an important role in moderating peer influences on substance use among disadvantaged, urban adolescents, and such moderation may differ according to an individual’s level of EF. This research provides evidence of differences in environmental susceptibility regarding contextual mechanisms of substance use among youth, and it informs the development of targeted substance use interventions that leverage social and environmental influences on adolescent substance use.

## 1. Introduction

Exposure to urban greenspace, such as street trees, parks, open space, and other vegetated or “nature” areas, is increasingly recognized as an important factor in the mental health of city residents [1,2,3]. Greenspaces have been associated with better self-reported health [4,5] and attention restoration [6,7]. Greenspace is also associated with the reduction of mental fatigue [8], lower levels of stress [7,9,10], reduced depression and anxiety [11,12], lower levels of crime, violence, and aggression [13,14,15], and reduced disease-based morbidity [4,5,16,17]. Greenspace at, or nearby, an individual’s residential location may play a particularly important role in mental health [16,17,18], particularly for youth and disadvantaged populations, who may have limited mobility and for whom local environmental characteristics are especially important [4,10,16].

The strength of the association between greenspace and mental health may differ based on individual characteristics, such as sex, race, ethnicity, or socioeconomic status [19,20]. Differences in the effects of greenspace may also be due to variation among individuals in the sensitivity to contextual and environmental effects. Individual variability in perceiving, processing, and responding to contextual demands is known as environmental sensitivity, which can be conceptualized on a continuum from low to high [21,22,23]. Importantly, environmental sensitivity has implications for both risky and protective influences on mental health and health behaviors, where greater sensitivity can amplify the developmental benefits or disadvantages conferred by protective or risky environmental characteristics, respectively. Executive function (EF), a construct capturing an interrelated set of cognitive skills and competencies connected to goal-directed behavior, including emotional regulation, planning, inhibition (i.e., impulse control), and attention, has been linked to sensitivity to contextual mechanisms, particularly among youth, where EF deficit (i.e., executive dysfunction) has been linked to greater susceptibility to peer and other contextual mechanisms of behavior [24,25].

The aim of the present study is to explore the role of residential greenspace exposure in substance use, an important mental health outcome and health behavior for which environmental context has been shown to play a key role [26] but which has been given little attention by greenspace or addiction researchers [27]. Our investigation focuses on a sample of urban adolescents, predominantly Black and economically disadvantaged, a population identified as particularly at risk for early initiation into substance use and substance use disorder [28,29]. In the present study, we examine the interaction of greenspace exposure with established social and psychological mechanisms which have been found to be related to adolescent substance use in previous research, specifically peer influence [30] and executive function [31,32].

We theorize that higher greenspace exposure may enhance the positive effects of pro-social peer influences on mitigating substance use through the attention restoration and stress reduction effects of exposure to green and natural environments [33,34,35,36,37], as well as through the positive effect of greenspace on enhanced social interaction [38]. We investigate whether the interactive effects of greenspace and peer influence differs between girls and boys, as our previous research suggests that contextual effects on adolescent substance use varies by sex [30,39]. We also investigate whether the interactive effects of greenspace and peer influence differs by level of EF, as evidence indicates that EF deficit is associated with adolescent substance use [31,40,41] and may increase sensitivity to social and environmental contextual characteristics associated with substance use [32].

While exploratory in nature, this study contributes to emerging research on greenspace and substance use within the context of the broader greenspace and mental health literature, and suggests how greenspace may be considered theoretically and analytically in combination with other more established social and psychological mechanisms of adolescent substance use.

## 2. Materials and Methods

### 2.1. Sample and Subject Recruitment

We utilize observations from a dataset of adolescent–parent pairs recruited from the pediatric emergency department of a hospital located in medium-sized city in the mid-Atlantic region of the U.S. that was part of a parent study investigating adolescent dating violence. Adolescents at the emergency department were being treated for medical treatment and were recruited by a nurse while awaiting treatment in a private room. Eligibility criteria include adolescents aged 14–17, accompaniment by a parent, fluency in English by both adolescent and parent, and adolescents not considered in acute medical distress as determined by the emergency department intake nurse. Recruitment occurred between April and November 2016 and took place during the work week (Monday–Friday) 9 a.m. to 5 p.m.

Following recruitment to the study by a nurse, a trained research assistant explained the data collection procedures to each adolescent and parent in detail and obtained written informed consent and assent from the adolescent. Each parent completed a short paper survey, and each adolescent completed a survey on a study laptop privately in the patient’s room with no one else present (including parents or medical staff). Because confidentiality is critical for collecting unbiased data, we made clear that the parent will not have access to the child’s data. Further, we provided private, separate settings so that the adolescent was not in the same room with the parent while he or she was completing the survey. Each parent received $10 and each adolescent received $20 to complete the assessment, an incentive structure informed by our previous research with this local population and consistent with common research practices to complete a one-time survey. The Virginia Commonwealth University Institutional Review Board approved the study protocol, and the National Institutes of Health provided a Certificate of Confidentiality. For more information on subject recruitment the reader is referred to Mason et al. (2020) [32].

### 2.2. Geocoding Home Address

The adolescent’s home address was collected in the parent survey and geocoded using the ArcGIS 10.6.1 (ESRI, Inc., Redlands, CA, USA) [42] geographic information systems (GIS) software package.

### 2.3. Demographics and Socioeconomic Status

Parents recorded the adolescent’s age (in years), sex (female or male), and race/ethnicity (encoded as Black versus not Black in the analysis because 81% of the sample identified as Black). Parents also recorded whether the student was eligible for free lunch at school (yes or no) as a measure of socioeconomic status.

### 2.4. Peer Network Health

Peer network health was measured using the Adolescent Social Network Assessment (ASNA) [43], an egocentric measure of the perceived behaviors and influences of an adolescent’s close peers. The assessment asks adolescents to provide information on three friends with whom they spend the most time with on average. For each friend, the adolescent reports negative or risky activities or behaviors, including substance use and participation in illegal, violent, and/or dangerous behaviors, as well as the degree to which the friend influences the adolescent to use substances. Adolescents are also asked about the prosocial activities associated with each friend, such as receiving help with school or emotional support. Scores for all friends are summed to yield a peer network health measure for each adolescent, where higher scores indicate a higher level of healthy peer network context. For more information on the ASNA the reader is referred to Mason et al. (2004) [43].

### 2.5. Substance Use

Substance use was captured in two stages. In the first stage, adolescents indicated whether they used alcohol, tobacco, illicit drugs, or medicine without a doctor’s prescription in the past two weeks using the Diagnostic Statistical Manual-5 (DSM-5) Level 1 screener for adolescents [44]. Adolescents who indicated substance use in the past two weeks were given the DSM-5 Level 2 substance use measure [45], which is intended to encode risk of problematic substance use. The measure is adapted from the National Institute on Drug Abuse-Modified ASSIST measure [46] and consists of frequency of use ratings on a five-point scale for various substances, which are then summed to yield a continuous substance use index score.

### 2.6. Executive Function

Executive function was measured using the Behavior Assessment System for Children, second edition, Parent Rating Scale (BASC-2 PRS) [47]. The BASC-2 EF content scale measures a parent’s perception of their child’s ability to plan and maintain goal-directed activity and to react appropriately to environmental feedback [47]. The measure is composed of 13 items where parents respond to each item on a four-point Likert scale. The raw score is converted to a T-score, which represents EF deficit where higher values indicate a greater deficit.

### 2.7. Greenspace

A common approach to measuring exposure to residential greenspace is the use of satellite imagery (e.g., Landsat imagery) to yield an index of vegetation, such as the normalized difference vegetation index (NDVI), as we have used in previous research [9]. While certainly a useful and valid measure of vegetation [48], limitations of this approach include the relatively coarse spatial resolution (e.g., 30 m pixel resolution) of easily accessible imagery, cloud contamination, and other uncertainties that disrupt the ability to capture the actual exposure to foliage experienced by individuals along the residential urban or suburban streetscape. For example, Liu et al. (2020) found that street view greenness was superior to greenness indices derived from satellite imagery in identifying associations of greenspace exposure with reduced risk of mental illness and increased sense of place attachment [49].

**Figure 1:**
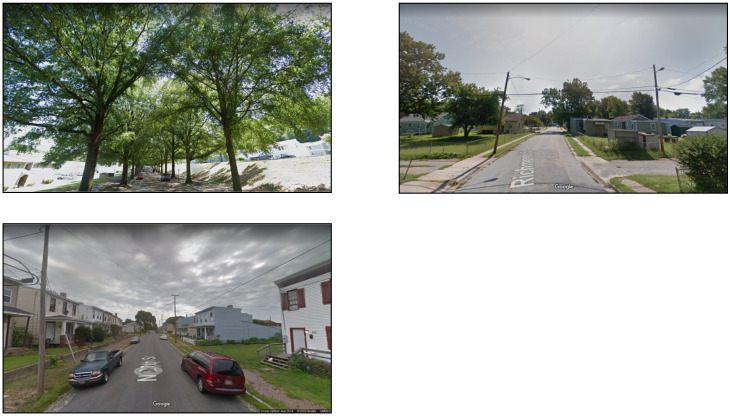
Examples of Google Street View images captured from residential locations in the study area where the GVI is more than one standard deviation above the mean (**top left**), at about the mean (**top right**), and more than one standard deviation below the mean (**bottom left**).

To capture the exposure to green vegetation at the streetscape level of each adolescent’s home address, geo-tagged Google Street View (GSV) images were used to quantify and map the amount of street greenery from the ground level. Unlike satellite imagery, which typically provides a coarse estimate of vegetation over a large area, GSV images have a similar view angle as compared to a person standing on the ground, and thus can be considered directly related to human perception of the surrounding environment [50,51]. In the present study we collected the nearest geo-tagged GSV images that were within 50 feet of each adolescent’s home address using the GSV image API [52]. The deep convolutional neural network PSPNet, trained based on ADE20K, was used to extract street greenery from the street-level images, where accuracy for the identification of greenery typically approaches 95% [53,54]. Based on the resulting image segmentation, we generate the green view index (GVI), which is calculated as follows:

(1) $$\mathrm{GVI}=\sum_{i=0}^{6}Area_{ri}/Area_{ti}\times 100\%$$

where the *Area_ri_* is the green (i.e., tree, shrub) pixel number in one of the six pictures taken in six different directions and * Area_ti_* is the number of total pixels in one of the six images [55]. The GVI therefore represents the visibility of the street greenery from a pedestrian’s perspective at each adolescent’s home location. For illustration purposes, Figure 1 shows examples of locations in the study area (but not at actual subject residences) where GVI is at the sample mean, below the mean, and above the mean.

### 2.8. Analytic Plan

Our sample for the present research consists of 126 adolescents for whom there were no missing data for the variables of interest. We began by generating descriptive statistics for all independent and dependent variables. We then tested three hypotheses:

**Hypothesis** **1.** *The association of peer network health with substance use is moderated by GVI, such that the effect of peer network health is stronger at higher GVI.*

**Hypothesis** **2.** *The moderating effect of GVI on the association of peer network health with substance use is moderated by sex, where the moderating effect is stronger for girls than boys.*

**Hypothesis** **3.** *The moderating effect of GVI on the association of peer network health with substance use is moderated by EF deficit, where the moderating effect is stronger for those with greater deficit.*

**Figure 2:**
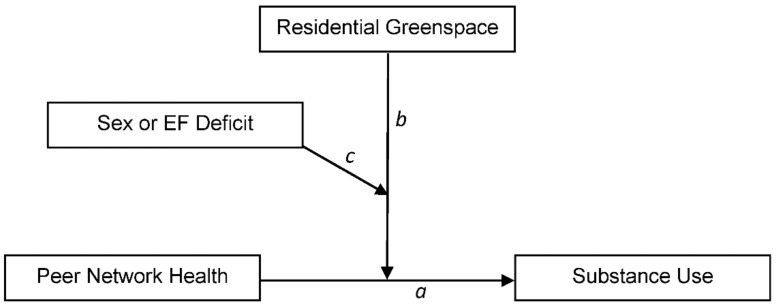
Conceptual diagram of moderated moderation models testing Hypotheses 2 and 3, where the direct effect of peer network health on substance use (path *a*) is moderated by residential greenspace exposure (GVI; path *b*), which in turn is moderated by sex or EF deficit (path *c*; Hypotheses 2 and 3, respectively).

To test Hypothesis 1 we first fit a linear regression model testing for the direct effects of peer network health and GVI on substance use, while controlling for age, sex, race, and whether the adolescent is eligible to receive free lunch at school. We then refit the model to include an interaction term consisting of the product of peer network health multiplied by GVI. To test Hypothesis 2 we entered a series of interaction terms for all two- and three-way combinations of the peer network health, GVI, and sex variables in a moderated moderation model, where the significance of the three-way interaction term indicates the presence of moderated moderation. To test Hypothesis 3 we refit an analogous model to that of Hypothesis 2, replacing the sex variable with the EF deficit variable. Figure 2 illustrates Hypotheses 2 and 3 diagrammatically.

All models were implemented in SPSS v. 27 (IBM, Armonk, NY, USA) [56]. Tests of moderation were implemented using the SPSS PROCESS package Models 1 (moderation) and 3 (moderated moderation) [57] using a 5000-sample bootstrap to estimate 95% confidence intervals for interaction terms. The PROCESS package probes the conditional effects of peer network health on substance use at three levels of the moderating term, GVI: the mean, one standard deviation above the mean, and one standard deviation below the mean. In the case of three-way interaction terms (as in the moderated moderation models), the conditional effects of peer network health are probed for all two-way combinations of the moderating terms at the mean, one standard deviation above the mean, and one standard deviation below the mean (for the continuous variable EF deficit) or at each categorical value (for the categorical variable sex). For all models, variables used to construct interaction terms were mean-centered prior to analysis to aid in interpretation.

## 3. Results

Table 1 provides descriptive statistics for the variables used in the analysis. The sample of 126 subjects is 60% male, 54% age 16–17, and 81% Black. Adolescents eligible to receive free lunch at school comprise 77% of the sample. Results of the direct effect and moderated regression models are reported in Table 2. Model 1 reports direct effects and indicates a significant (*p* < 0.05) negative relationship between peer network health and substance use; as expected, higher peer network health is associated with a lower level of substance use.

**Figure 3:**
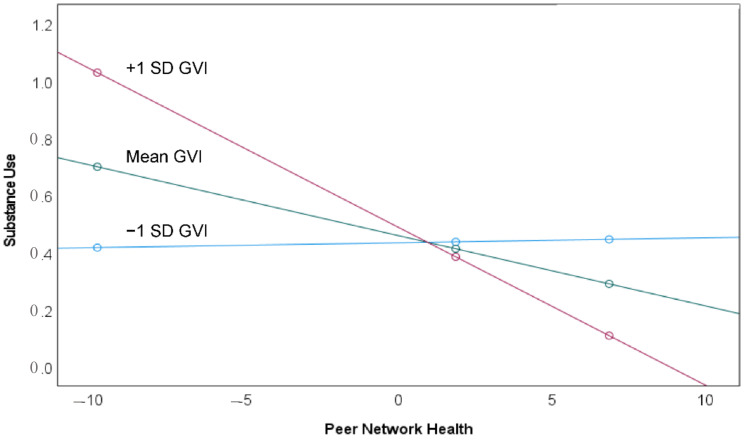
The moderating effect of GVI on the association of peer network health with substance use, where the association differs at locations where GVI is one standard deviation below the mean (blue), at the mean (green), and one standard deviation above the mean (red). The effect of peer network health on substance use is significant for adolescents where residential GVI is at or above the mean, but not where residential GVI is low.

Model 2 (Table 2) reports the results of the moderation of the effect of peer network health on substance use by greenspace exposure. The interaction term is significant (*p* < 0.05), indicating that Hypothesis 1 is supported. The conditional effect of peer network health on substance use is significant (*p* < 0.05) and negative when GVI is at the mean and one standard deviation above the mean, but not at lower levels of GVI (Table 3). Thus, the negative (i.e., mitigating) effect of peer network health on substance use is enhanced at higher levels of greenspace exposure. This is illustrated graphically in Figure 3, where the association of peer network health with substance use has a steep and negative slope at values of GVI at the mean and one standard deviation above the mean. At GVI values one standard deviation below the mean, the slope of the peer network health/substance use association is near zero.

Model 3 (Table 2) reports the results of the three-way moderation of peer network health, greenspace exposure, and sex. The three-way interaction term is not significant (*p* = 0.65), indicating that Hypothesis 2 is not supported. The moderating effect of GVI on the association between peer network health and substance use does not differ significantly between girls and boys.

**Figure 4:**
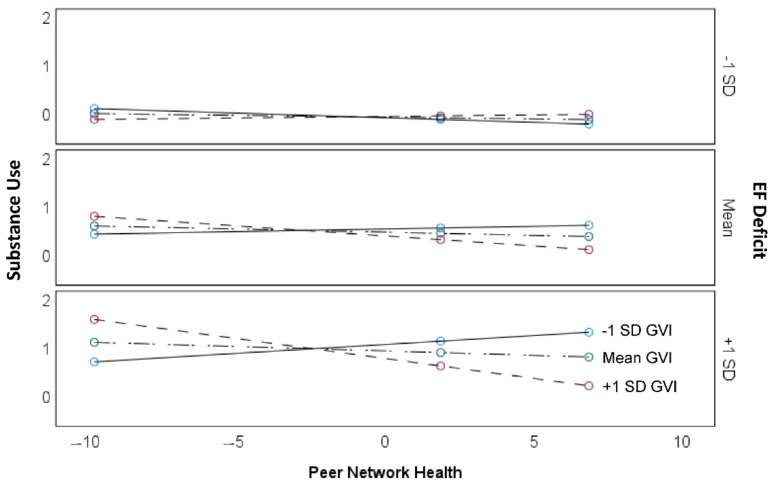
The moderating effect of GVI on the association of peer network health with substance use at different levels of executive functioning (EF). The bottom panel shows that for adolescents with a high level of EF deficit, GVI significantly moderates the association of peer network health with substance use, where at higher levels of GVI (short dashed line with red markers) the association is significant and negative. Whereas, when EF deficit is high, the peer network health-substance use association is not significant at mean (long dashed line with green markers) and lower levels (solid line with blue markers) of GVI. For adolescents with low EF deficit (top panel), there is no significant moderating effect of GVI on the association of peer network health with substance use.

Model 4 (Table 2) reports the results of the three-way moderation of peer network health, greenspace exposure, and EF deficit. The three-way interaction term is significant (*p* < 0.05), indicating that Hypothesis 3 is supported. The conditional effect of peer network health on substance use is significant (*p* < 0.05) and negative when greenspace exposure is one standard deviation above the mean and EF deficit is at the mean or higher (Table 4). In other words, in this sample, the moderating effect of greenspace exposure on the association of peer network health with substance use occurs among youth with average to high EF deficit. Figure 4 illustrates this pattern, where the greatest differentiation in the association of peer network health with substance use at different levels of greenspace exposure occurs where EF deficit is one standard deviation above the mean (Figure 4, bottom panel).

## 4. Discussion

To our knowledge, this is the first study to investigate the interactive effects of greenspace on the role of peers, sex, and EF in adolescent substance use. Our results suggest that residential greenspace interacts with peer network characteristics, such that the prophylactic effects of prosocial peers on substance use occurs in concert with greener residential environments. These interacting peer/place contextual mechanisms of substance use behavior are activated particularly for adolescents with higher EF deficits, who may be considered to have increased susceptibility to contextual social and environmental influences on substance use behaviors. We did not find that the interacting effects of greenspace and peer network health differed by sex; rather, they were similar among girls and boys.

Our results are consistent with previous research on the association of prosocial peers with reduced substance use [29,30], as well with previous research on the association of greenspace exposure with better mental health outcomes [11,12]. We speculate that increased exposure to green vegetation along the streetscape at an adolescent’s residence reduces mental fatigue and enhances attention restoration [2,8], though research also suggests related biophysical mechanisms such as exposure to air pollution may play a role [58]. Our results also suggest that the positive or restorative effects of greenspace may operate differentially contingent on other contextual factors. In neighborhoods with average or greater greenspace, adolescents in close peer networks that are inclined toward engaging in prosocial activities and toward providing and receiving emotional support may use the natural positive and restorative aspects of greenspace to engage in health promoting activities. Subjects with more healthy peer networks may be more likely to derive the positive psychological aspects of greenspace as well as engage in physical activity which may help reduce stress and further enhance positive psychological states, thus facilitating an increased effect of positive peer network health on substance use behavior.

A greener streetscape may also encourage greater social interaction with peers [16,17], particularly among disadvantaged youth for whom there may be fewer opportunities to leave their residential neighborhood [4,10], which may translate into greater peer effects on substance use behaviors. Alternatively, it may be the case that peer network health and greenspace exposure work in concert to influence substance use, where it is the accumulative positive effects of healthier peer networks and higher greenspace exposure that lead to reduced substance use. At the same time, our results suggest that neighborhoods with average or greater greenspace, when coupled with peer networks characterized by negative or risky behaviors, may actually facilitate substance use. Rather than using neighborhood greenspace for positive activities that are physically and psychologically enhancing, unhealthy peer networks may encourage the use of greenspaces such as parks for illicit activities like substance use, though we emphasize that these interpretations are speculative.

Our findings regarding EF are also consistent with dynamic systems theories of resilience [59], adolescent neurobiological development [60], and research on differential susceptibility [21,23]. Individual characteristics such as EF may impart differential sensitivity to environmental influences, which may be due in part to brain development, such that EF deficit may heighten vulnerability in risky contexts while also strengthening responses to protective experiences. Our results highlight the way that EF capacity may create differential susceptibility to the influence of peers, greenspace, or their interactions.

Epidemiologic studies indicate that the effect of natural environments on the restoration of cognitive and physiological capacities (such as attention and physiological stress reactivity) and psychological and social capacities [61] are intertwined, and that the association between urban greenspace exposure and young adult mental and behavioral health is mediated sequentially by restoring attention and then building mindfulness and reducing rumination [62]. Each step in this sequence reflects, in part, higher-order control of directed and sustained attention, one of the key EF abilities [63]. These linkages are not unexpected, as EF is linked with greater mindfulness empirically [64,65], and mindfulness training influences growth in EF skills [66] as well as changes in the brain regions associated with EF skills [67]. Mindfulness-based interventions are effective methods of reducing the frequency and quantity of alcohol and drug use, substance-related problems, and cravings [68]. Moreover, improvements in EF achieved in mindfulness programs can improve functioning in daily activities [69]. Promising data show that mindfulness programs may also be an effective substance use prevention for children and youth [70].

Most mindfulness training programs primarily involve focused-attention exercises that require cognitive effort but relaxed alertness to suppress mind-wandering and distractions [71]. These exercises can be effective but are often challenging for individuals with poorer attentional capacities. In contrast, open-monitoring exercises rely on minimal effort to connect with internal and external experiences [71] and have been used effectively in programs such as Restoration Skills Training (ReST) that are purposefully conducted in a natural environment [72]. Nature environments promote ‘soft-fascination’ [36] which may enhance open-monitoring training but may also organically facilitate these neurodevelopmental processes. In the present study, it may be that adolescents with higher EF deficit benefit more from greenspaces’ inherent soft-fascination characteristics, which draw attention softly and effortlessly. That is, adolescents residing in high greenspace streetscapes may gain in protection specifically because they do not have to draw on higher order focused attentional capacities.

Given that this sample of adolescents and their families are likely experiencing greater than average levels of psychological stress due to the multi-prong effects of poverty, the findings that greater levels of residential greenspace exposure coupled with pro-social behavior of peers has a protective effect against substance use is promising, and is consistent with research that posits differential susceptibility to environmental contexts within an adaptive/evolutionary model [21,23,32,73]. Thus, an adolescent who has high EF deficit due to living in a disadvantaged, high stress environment may be more sensitive to the benefits of greenspace exposure and prosocial peer influence regarding substance use. For some adolescents, exposure to stressful environments can improve various forms of attention, perception, learning, and memory [23,73], what Ellis et al. (2020) refer to as “hidden talents” (i.e., enhanced adaptive problem-solving skills developed as a response to adverse environments) [74]. While the current study does not directly address the association of environmental factors with such “hidden talents”, our findings provide support for differential susceptibility among low-resource urban youth, highlight the ways in which protective environmental and social factors can be leveraged for youth living in potentially high-stress contexts, and thus contribute to the development of contextually sensitive substance use interventions.

We acknowledge several limitations to the study. First, the cross-sectional design limits inference as to causal relationships among variables. In addition, this study analyzes a relatively small sample of convenience of adolescents recruited from a single site, and therefore the generalizability of our findings is limited. We further acknowledge that our sample consists of only youth who had parents willing and able to accompany them to the emergency department, which may restrict the sample to certain groups of adolescents, such as those with parents present and willing to participate in the study; It may be possible that such adolescents are less likely than others to use substances. The nonrandom nature of the sample warrants emphasizing the exploratory nature of our investigation.

Our measure of substance use, while informed by DSM-5 criteria, is a general measure of substance use severity and does not distinguish between different types of substances, which vary in potential harms. The most common substance used in the sample is marijuana, which may confer a lesser risk than other ‘harder’ drugs such as cocaine and opioids. Poly-substance use is also an important consideration, where the use of multiple substances is associated with greater health risk. Investigating the relationships among peer network health and greenspace with regards to different types and combinations of substance use represents an important future research topic.

Our measure of EF deficit relies on parent reports. Given the subject recruitment setting (hospital emergency department), assessment battery length was an important consideration. Because of this, and prior research that supports parental reporting, we decided that having parents report on their children’s EF was the most efficient method to collect objective EF data. However, direct adolescent measures of EF deficit may have yielded more accurate assessments. We also acknowledge that our substance use measure, which is based on adolescent self-report, may be biased if subjects were reluctant to report substance use for fear of getting into trouble. There are also other potential influences on adolescent substance use, such as access to substances, family history of substance use, and psychological trauma, that are unaccounted for in our models.

Additionally, this study lacks information about how adolescents actually experience and use greenspaces for certain activities. Certainly, the activities and social interactions that occur at particular locations confer meaning and emotional value, from which stems place influence on health and health behaviors. Understanding the psychological expectations that are likely driven by the unique place-based social dynamics experienced by each adolescent would provide insights into place-based mechanisms of behaviors such as substance use.

Another potential limitation concerns the role of neighborhood socioeconomic disadvantage, which has been found to be negatively correlated with greenspace [75], and thus may act as a confounder in our models of substance use. While our models include eligibility to receive free lunch at school as a measure of individual level economic disadvantage, we acknowledge this may not capture neighborhood level disadvantage. As a post hoc sensitivity test we refit the models presented in Table 2 by also including a U.S Census Bureau tract-level measure of socioeconomic disadvantage [76] which we have used in previous research [77,78], calculated as $\left(\left(\left(a/10\right)+\left(b/10\right)\right)-\left(\left(c/10\right)+\left(d/10\right)\right)\right)/4$, where *a* is the percentage of households with income below the poverty level, *b* is the percentage of female-headed households with children, *c* is the percentage of adults 25 years or older with a bachelor’s degree or higher, and *d* is the percentage of owner-occupied housing units. Variables are derived from annual American Community Survey, U.S. Census Bureau data. Higher values indicate greater socioeconomic disadvantage. Model results were substantially the same as those reported above, with no differences in the significance (*p* < 0.05) or direction of any observed moderating effects; consequently, results of the sensitivity analysis did not alter our conclusions.

## 5. Conclusions

Though we emphasize that the present research is exploratory and our interpretations are speculative, these results suggest that greenspace exposure may play an important role in moderating peer influences on substance use among disadvantaged, urban adolescents, and that the susceptibility to such environmental and social factors may differ according to an individual’s level of EF. Such findings can inform further investigations into the development of substance use interventions that leverage the role of environmental and peer mechanisms of substance use among youth and target adolescents who may be particularly sensitive to such contextual interventions. Future research should (i) examine the effect of greenspace exposure on stress, anxiety, depression, and other psychological states and mood disorders; (ii) examine differences in effects by sex [79] and their consequent impact on adolescent substance use using more rigorous, experimental study designs [80]; and (iii) evaluate the efficacy of interventions that implement peer- and place-oriented strategies to reduce adolescent substance use.

## Figures and Tables

**Table 1:** Descriptive Statistics (*n* = 126).

Variable	Values		Frequency	Percent
Sex	Female		76	60.3%
	Male		50	39.7%
Age	14		27	21.4%
	15		31	24.6%
	16		31	24.6%
	17		37	29.4%
Race	Black		102	81.0%
	Other		24	19.0%
Free Lunch	Yes		97	77.0%
	No		29	23.0%
**Variable**	**Min**	**Max**	**Mean**	**SD**
Substance Use	0.00	13.00	0.40	1.39
Peer Network Health	−22.00	42.00	17.13	11.37
GVI	2.84	82.95	37.49	16.20
EF Deficit	33.00	99.00	53.34	12.78

**Table 2:** Results of regression models.

Variable	Model 1 ^a,b^	Model 2 ^a,b^	Model 3 ^a,b^	Model 4 ^a,b^
Age	0.127 (−0.096, 0.349)	0.138 (−0.081, 0.357)	0.132 (−0.096, 0.360)	0.116 (0.054, 0.366)
Sex (Female = 1, Male = 0)	−0.234 (−0.733, 0.265)	−0.246 (−0.737, 0.246)	−0.208 (−0.713, 0.297)	−0.267 (−0.734, 0.201)
Race (Black = 1, Other = 0)	−0.202 (−0.954, 0.550)	−0.152 (−0.894, 0.590)	−0.082 (−0.838, 0.673)	0.015 (−0.695, 0.725)
Free Lunch (Yes = 1, No = 0)	0.158 (−0.531, 0.846)	0.158 (−0.520, 0.836)	0.160 (−0.534, 0.853)	0.009 (−0.643, 0.661)
Peer Network Health (PNH)	−0.026 * (−0.048, −0.004)	−0.027 * (−0.049, −0.005)	−0.007 (−0.044, 0.030)	−0.016 (−0.037, 0.006)
Greenspace (GVI)	0.001 (−0.015, 0.016)	0.002 (−0.014, 0.017)	−0.011 (−0.037, 0.015)	−0.005 (−0.019, 0.010)
PNH × GVI		−0.002 * (−0.003, 0.000)	−0.001 (−0.001, 0.002)	−0.002 * (−0.003, 0.000)
PNH × Sex			−0.031 (−0.076, 0.015)	
GVI × Sex			0.019 (−0.014, 0.052)	
PNH × GIVI × Sex			−0.001 (−0.004, 0.003)	
Executive Function Deficit (EFD)				0.038 *** (0.018, 0.058)
PNH × EFD				−0.001 (−0.003, 0.001)
GVI × EFD				−0.0004 (−0.002, 0.001)
PNH × GVI × EFD				−0.0002 * (−0.0003, 0.0000)

Note: ^a^ Coefficients reported, 95% CI in parentheses; ^b^ * *p* < 0.05, *** *p* < 0.005.

**Table 3:** Conditional effects of peer network health on substance use at the mean and +/−1 standard deviation of GVI.

GVI Value	Effect ^a,b^	Lower 95% CI	Upper 95% CI
−1 SD	0.002	−0.032	0.035
Mean	−0.025 *	−0.046	−0.003
+1 SD	−0.055 ***	−0.089	−0.021

Notes: ^a^ Coefficients reported, ^b^ * *p* < 0.05, *** *p* < 0.005.

**Table 4:** Conditional effects of peer network health on substance use at the mean and +/−1 standard deviation of GVI and EF deficit values.

GVI Value	EF Deficit Value	Effect ^a,b^	LL 95% CI	UL 95% CI
−1 SD	−1 SD	−0.019	−0.062	0.024
−1 SD	Mean	0.011	−0.021	0.043
−1 SD	+1 SD	0.037	−0.011	0.085
Mean	−1 SD	−0.007	−0.040	0.026
Mean	Mean	−0.013	−0.035	0.008
Mean	+1 SD	−0.018	−0.048	0.012
+1 SD	−1 SD	0.006	−0.054	0.067
+1 SD	Mean	−0.041 *	−0.075	−0.008
+1 SD	+1 SD	−0.082 ***	−0.125	−0.038

Notes: ^a^ Coefficients reported, ^b^ * *p* < 0.05, *** *p* < 0.005.

## Data Availability

Individual level data are protected by confidentiality as participants were minors and sensitive data were collected.
